# Conventional vs Augmented Reality–Guided Lateral Calcaneal Lengthening Simulated in a Foot Bone Model

**DOI:** 10.1177/10711007241237532

**Published:** 2024-03-19

**Authors:** Maksym Polt, Arnd F. Viehöfer, Fabio A. Casari, Florian B. Imhoff, Stephan H. Wirth, Stefan M. Zimmermann

**Affiliations:** 1 Department of Orthopaedics, Balgrist University Hospital, University of Zürich, Zürich, Switzerland

**Keywords:** Hintermann osteotomy, Acquired adult flatfoot deformity, Augmented reality, HoloLens, Lateral column lengthening osteotomy, Calcaneus, Subtalar facets, Surgery, Osteotomy

## Abstract

**Background:**

Acquired adult flatfoot deformity (AAFD) results in a loss of the medial longitudinal arch of the foot and dysfunction of the posteromedial soft tissues. Hintermann osteotomy (H-O) is often used to treat stage II AAFD. The procedure is challenging because of variations in the subtalar facets and limited intraoperative visibility. We aimed to assess the impact of augmented reality (AR) guidance on surgical accuracy and the facet violation rate.

**Methods:**

Sixty AR-guided and 60 conventional osteotomies were performed on foot bone models. For AR osteotomies, the ideal osteotomy plane was uploaded to a Microsoft HoloLens 1 headset and carried out in strict accordance with the superimposed holographic plane. The conventional osteotomies were performed relying solely on the anatomy of the calcaneal lateral column. The rate and severity of facet joint violation was measured, as well as accuracy of entry and exit points. The results were compared across AR-guided and conventional osteotomies, and between experienced and inexperienced surgeons.

**Results:**

Experienced surgeons showed significantly greater accuracy for the osteotomy entry point using AR, with the mean deviation of 1.6 ± 0.9 mm (95% CI 1.26, 1.93) compared to 2.3 ± 1.3 mm (95% CI 1.87, 2.79) in the conventional method (*P* = .035). The inexperienced had improved accuracy, although not statistically significant (*P* = .064), with the mean deviation of 2.0 ± 1.5 mm (95% CI 1.47, 2.55) using AR compared with 2.7 ± 1.6 mm (95% CI 2.18, 3.32) in the conventional method. AR helped the experienced surgeons avoid full violation of the posterior facet (*P* = .011). Inexperienced surgeons had a higher rate of middle and posterior facet injury with both methods (*P* = .005 and .021).

**Conclusion:**

Application of AR guidance during H-O was associated with improved accuracy for experienced surgeons, demonstrated by a better accuracy of the osteotomy entry point. More crucially, AR guidance prevented full violation of the posterior facet in the experienced group. Further research is needed to address limitations and test this technology on cadaver feet. Ultimately, the use of AR in surgery has the potential to improve patient and surgeon safety while minimizing radiation exposure.

**Clinical Relevance:**

Subtalar facet injury during lateral column lengthening osteotomy represents a real problem in clinical orthopaedic practice. Because of limited intraoperative visibility and variable anatomy, it is hard to resolve this issue with conventional means. This study suggests the potential of augmented reality to improve the osteotomy accuracy.

## Graphical abstract

**Graphical Abstract:**
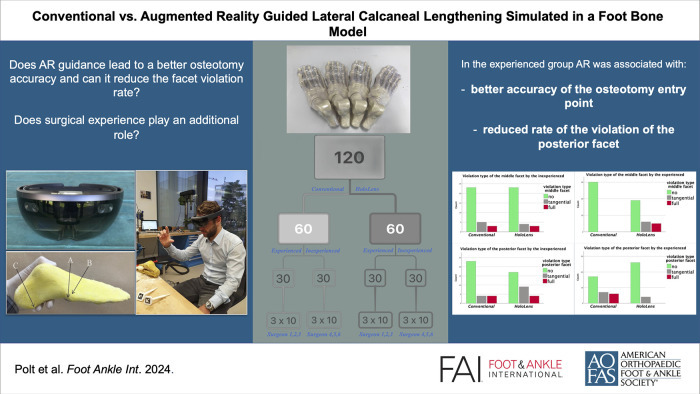
This is a visual representation of the abstract.

## Introduction

**Figure 1.**
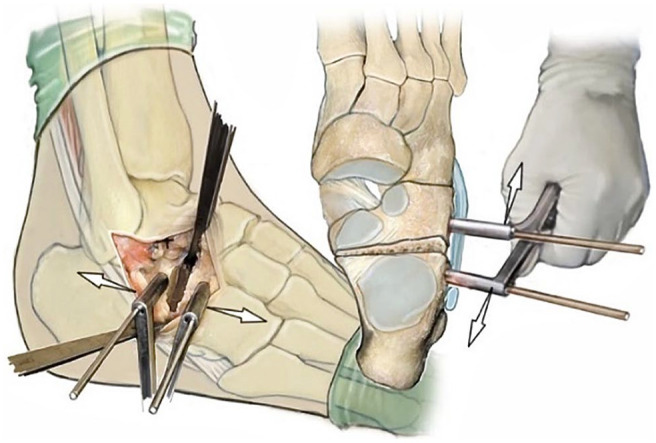
Hintermann osteotomy with a starting point 10-15 mm proximal to the calcaneocuboid joint.^12^

Stage II acquired adult flatfoot deformity (AAFD), considered flexible,^15,21^ can be treated with reconstruction of medial soft tissue structures combined with lateral column lengthening osteotomy of the calcaneus.^1,6,22,25^ This osteotomy, known as the Hintermann osteotomy (H-O) (Figure 1), is a joint-preserving procedure that has been modified over the years to correct acquired flatfoot deformity in adults.^8,13,20^

Anatomical studies have revealed variations in the subtalar facets, with type A and B being equally common and type C being rare (Table 1).^3,23^ The H-O is meant to pass through the sinus tarsi and between the middle and posterior facets to avoid iatrogenic facet violation.^13^ However, placement between the 2 facets can be challenging because of anatomical variations and limited intraoperative visibility. Thus, violation of the middle or posterior facets remains a possible complication of the H-O.^7,12^ From the biomechanical standpoint, it is especially crucial to prevent injury of the posterior facet, because of its role as a major loadbearing area.^10^

**Table 1.** Modified Classification of the Subtalar Articular Facets by Bunning & Barnett.^3,23^

Type A	3 facets
A1	Three distinct facets
A2	Confluence of middle and anterior facets, which remains clearly distinct through the presence of a ridge between them
Type B	2 facets
B1	Fusion of middle and anterior facets with narrowing at the site of the fusion
B2	Complete fusion of middle and anterior facets
B3	The anterior facet is missing
Type C	Fusion of all 3 facets

**Figure 2.**
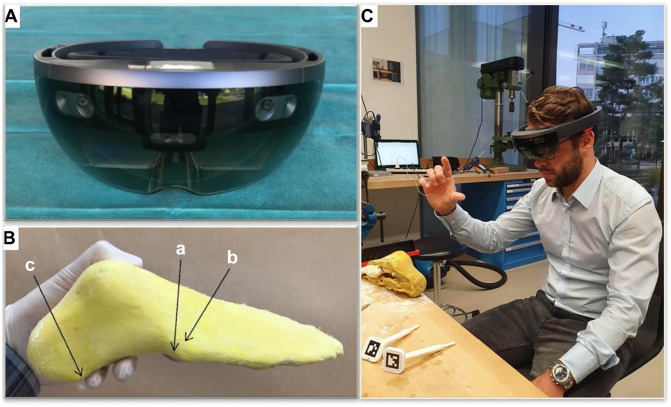
(A) Microsoft HoloLens 1 headset. (B) Foot model with lateral surgical landmark: base of os metatarsale V (a), the superolateral osteophyte on the base of os metatarsale IV (b), and processus lateralis tuberis calcanei (c). (C) Orthopaedic surgeon starting the registration of the hologram.

In an effort to improve the accuracy of the H-O, we tested the application of augmented reality (AR) using the Microsoft HoloLens headset (Figure 2A). The HoloLens is a mixed reality headset that allows for the projection of high-quality 3D holograms into the real-world environment.^18^ When wearing the HoloLens, one can superimpose and precisely align holograms to specific anatomic structures (Figure 2C). The interest for AR in orthopaedic surgery has been growing from proof of concept^2,4,5,11,16,24^ to cadaver studies^14,17^ and to in situ AR pedicle screw navigation.^9^

We aimed to assess the feasibility and accuracy of AR-guided calcaneal osteotomy compared to conventional surgery. Our objectives were to explore whether this new technology could improve the accuracy of the specific osteotomy and reduce the facet violation rate, as well as to investigate how factors such as surgical experience might influence the results of AR-assisted surgery. We hypothesized that use of AR guidance would improve the accuracy of an experienced as well as inexperienced surgeon, with the effect being more pronounced in inexperienced surgeons.

## Materials and Methods

### Study Setup

The experiment was conducted using 120 identical right foot bone models (Synbone 9110, Synbone AG, Zizers, Switzerland), with 60 undergoing osteotomies using augmented reality (AR) guidance, and the other 60 undergoing osteotomies in a control group without AR guidance. Foot bone models were encased in a replaceable soft tissue cover consisting of a solidified latex emulsion (Latexemulsion 500 mL; Rayher, Hobby GmbH, Laupheim, Germany) (Figure 2B). This soft tissue cover precisely mimicked the actual skin, covering the bony structures with a thin latex layer and allowing the surgeon to palpate the relevant surgical landmarks of the lateral hindfoot and midfoot demonstrated in Figure 2B.

Six surgeons (3 experienced vs 3 inexperienced) each performed 10 conventional and 10 AR-assisted osteotomies under identical conditions. None of the 6 surgeons had prior experience in AR.

In order to avoid a learning effect and thus possible distortion of the results, the osteotomies were performed in the following manner: first the conventional osteotomies were performed during 3 different days with 1-week breaks between those days, with surgeons switching after each osteotomy with small breaks in between the procedures, then the AR-assisted osteotomies were performed in the same manner. A maximum of 4 osteotomies were performed by each surgeon per day in order to minimize adaptation to the setting. No direct evaluation of the performed osteotomy was allowed.

### Augmented Reality

**Figure 3.**
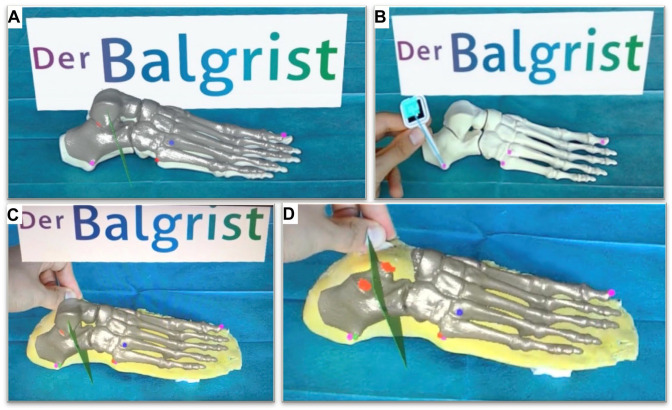
(A) 3D foot hologram projected on the foot bone dummy using HoloLens 1 headset. (B) Marking the processus lateralis tuberis calcanei as point 3. (C) 3D foot hologram projected on the foot model using HoloLens 1 headset. (D) The most optimal view of the cutting plane in green with hidden talus and market facets (red). [See online article for color figure.]

A computed tomography scan (Philips Brilliance 40 CT; Philips Healthcare, the Netherlands) of the foot bone model was acquired and the DICOM file was uploaded into the image processing tool (Materialise Mimics Medical 19.1; Materialise, Leuven, Belgium) for density-based segmentation. This segmentation generated a 3D image of the foot model, which was then exported into an in-house surgical planning software (CASPA, version 5.29; Medacta, Castel San Pietro, Switzerland). The 3D image was then reviewed by senior foot and ankle surgeons, who determined the ideal cutting plane for the Hintermann osteotomy. The starting point was placed 11.5 mm proximal to the calcaneocuboid joint and was designed to run through the sinus tarsi, without damaging the middle and posterior facets. The 3D image of the foot model was then uploaded into a HoloLens 1 (Microsoft Corporation, Redmond, WA) headset using a HoloLens support tool (Unity, Unity Technologies, San Francisco). This resulted in a high-resolution 3D foot hologram, including the computer-planned cutting plane, which was projected into the HoloLens user’s visual field (Figure 3A). The previously mentioned landmarks of the lateral hindfoot and the distal end of the great toe phalanx were used to align the HoloLens foot hologram with the foot models by marking the landmarks in a specific order using the in-house surgical planning software (CASPA, version 5.29; Medacta) and a custom-made pointing device recognized by the HoloLens (Figure 3B). The preoperatively planned hologram with the ideal cutting plane was precisely superimposed with the foot model, and specific voice commands were used to make changes to the properties of the hologram, such as hiding or unhiding the talus and marking or unmarking the facets (Figure 3C). The setup with hidden talus and marked posterior and middle facets provided the optimal view of the osteotomy plane (Figure 3D).

### Conventional Osteotomy

**Figure 4.**
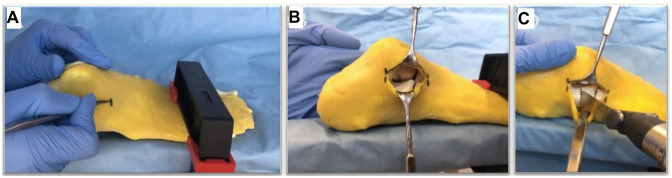
(A) Soft tissue incision, (B) view over the sinus tarsi, and (C) osteotomy starting point.

A horizontal soft tissue incision extending from the distal aspect of the fibula to the anterior process of the calcaneus afforded access to the sinus tarsi (Figure 4A and B). In each instance, the surgeon aimed to initiate the incision 11.5 mm proximal to the calcaneocuboid joint, which was also marked as the starting point on the AR hologram (Figure 4C). The osteotomy was executed using a 1506.1 oscillating saw blade (Sodem Systems, Geneva, Switzerland) and completed through the medial cortex. No bone chisel was used because of insufficient stiffness of the foot model and thus danger of breakage and imprecise osteotomy.

### AR Osteotomy

Following the identical soft tissue incision, the first step of the AR-guided procedure involved marking the prespecified foot landmarks using a commercially available sterile fiducial marker (Clear Guide Medical, Baltimore, MD). The exact position of the pointing device was detected and the landmarks were saved, enabling automatic hologram alignment. The projected hologram not only visualized the hindfoot structures but also provided an ideal 3D cutting plane for the H-O. This cutting plane served as the basis for determining the starting point and direction of the osteotomy. Although surgeons were granted the opportunity to reregister the hologram for alignment purposes, they were expected to strictly adhere to the holographic cutting plane once satisfactory alignment was achieved. The osteotomy was then performed in the same manner as the conventional procedure.

### Evaluation of Osteotomy Precision

To assess the accuracy of the osteotomies performed, the posterior and middle facets within the region of the osteotomy were marked using a permanent marker. To avoid measurement bias, a new numbering system was implemented for the osteotomized calcaneal bones, and the photo documentation and accuracy measurements were conducted by an individual separate from the surgical component of the study.

**Figure 5.**
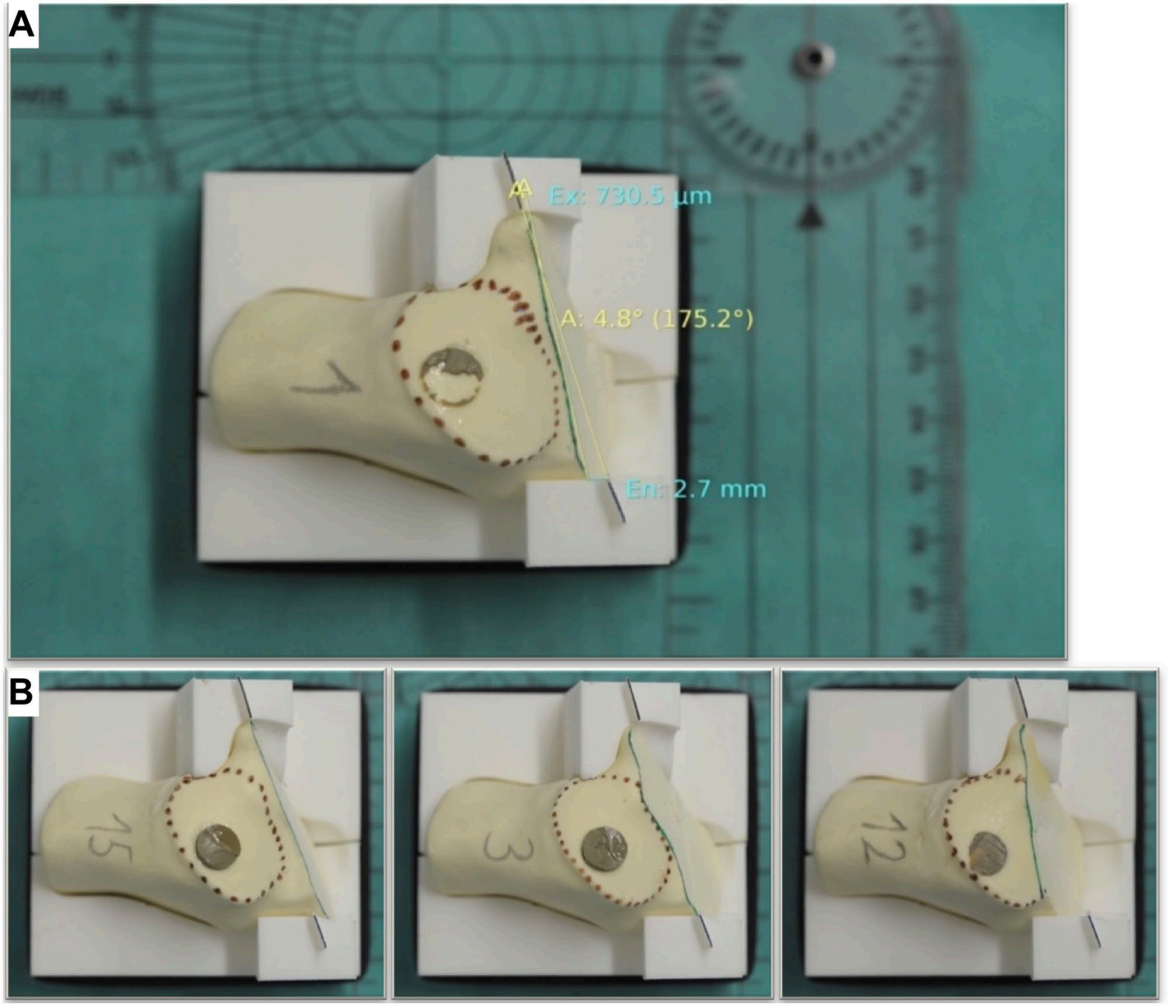
(A) Digital measurement of the osteotomy precision. (B) No violation (left), tangential violation (center), and full violation (right).

Standardized and reliable photo documentation was facilitated through the use of 3D-printed surgical guides. Using the in-house surgery planning software, CASPA (version 5.29; Medacta), 2 guides were created from a previously generated 3D foot model image, with their inner surfaces shaped to match the medial and lateral surfaces of the calcaneus (Figure 5A). These highly precise guides enabled consistent and reproducible evaluations of the performed osteotomy and its deviation from the ideal cutting plane, including the distance to the ideal starting and exit points (Figure 5A).

Each of the 120 osteotomized calcaneal bones were positioned within the 3D-printed guides and placed on an even surface beneath a fixed camera (Nikon D7000 DSLR Camera; Nikon, Tokyo, Japan). The photos were then imported into the radiologic software, Synedra (Synedra Information Technologies GmbH, Innsbruck, Austria), for measurements.

The accuracy of each performed osteotomy was compared to the ideal osteotomy line drawn between the ideal entry and exit points on the 3D-printed guides (Figure 5A). The following parameters were evaluated:

- The deviation of the entry and exit points of the performed osteotomies from the ideal line in millimeters in the anterior or posterior direction.
- The deviation of the performed osteotomy from the ideal line in degrees in the anterior or posterior direction.

Additionally, the integrity of the middle and posterior facets, the most endangered articular structures during the H-O, was evaluated using the following classification system (Figure 5B):

- No violation
- Tangential violation
- Full violation

The following subgroups were compared to assess the accuracy of the calcaneal osteotomies:

- All conventional vs all AR-guided osteotomies
- Conventional vs AR-guided osteotomies performed by experienced surgeons
- Conventional vs AR-guided osteotomies performed by inexperienced surgeons
- Facet violation in all conventional vs facet violation in all AR-guided osteotomies
- Facet violation in conventional vs facet violation in AR-guided osteotomies performed by inexperienced surgeons
- Facet violation in conventional vs facet violation in AR-guided osteotomies performed by experienced surgeons
- Facet violation in conventional osteotomy performed by experienced vs inexperienced surgeons
- Facet violation in AR-guided osteotomy performed by experienced vs inexperienced surgeons

### Statistics

Statistical analysis was performed using IBM SPSS Statistics (version 26; IBM Corp, Armonk, NY). All numeric data were reported as mean (SD) and 95% CI. The data was tested for normality. On determining the absence of a normal distribution, a Mann-Whitney *U* test was employed to evaluate the numeric data. For the analysis of the facet violation, a combination of the chi-square test and Fisher exact test was used, with the latter being employed in cases where the expected count of cells was less than 5 or the sample size was less than 40. The *P* value of <.05 was considered significant. In the event of statistically significant results, a post hoc analysis was conducted to identify the variable responsible for the observed differences.

## Results

### Osteotomy Accuracy

**Figure 6.**
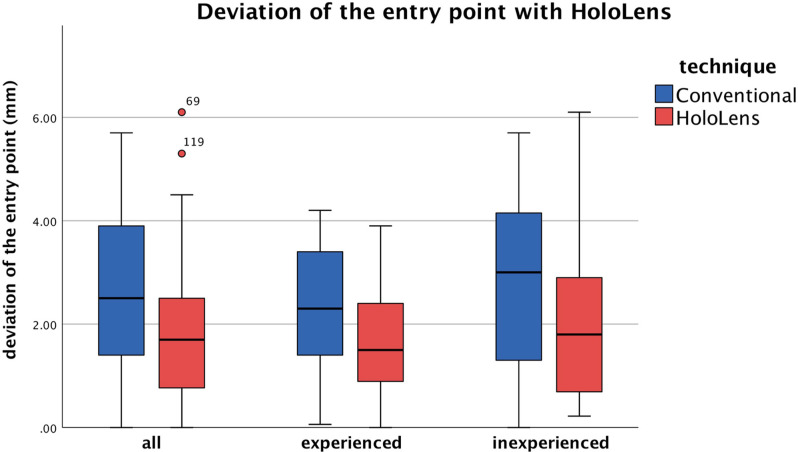
Application of HoloLens led to improved precision of the osteotomy entry point, with statistically significant difference in the experienced group.

AR-guided osteotomies exhibited a statistically significant improvement in the accuracy of the osteotomy entry point compared with the conventional method (*P* = .004). The mean deviation from the predefined ideal entry point, located 11.5 mm proximal to the calcaneocuboid joint, was found to be 1.8 ± 1.3 mm (95% CI 1.48, 2.12) in the AR-guided group, whereas in the conventional group, it was 2.5 ± 1.5 mm (95% CI 2.17, 2.91). Further analysis revealed that the experienced surgeons exhibited a greater degree of accuracy in placement of the blade using AR guidance with a mean deviation of 1.6 ± 0.9 mm (95% CI 1.26, 1.93) compared to 2.3 ± 1.3 mm (95% CI 1.87, 2.79) in the conventional method (*P* = .035). The inexperienced surgeons also demonstrated an improved accuracy of the osteotomy entry point with the mean deviation of 2.0 ± 1.5 mm (95% CI 1.47, 2.55) using AR guidance compared with 2.7 ± 1.6 mm (95% CI 2.18, 3.32) in the conventional method, although this difference was not statistically significant (*P* = .064) (Figure 6).

Analysis of the deviation of the exit point revealed no statistically significant differences between the AR-guided and conventional methods for either experienced or inexperienced surgeons. However, it is worth noting that the experienced surgeons exhibited a higher mean deviation of the exit point of 3.2 ± 2.6 mm (95% CI 2.31, 4.14) with AR guidance compared with 2.2 ± 1.8 mm (95% CI 1.51, 2.82) in the conventional method, although this difference was not statistically significant (*P* = .13). No statistically significant differences were found in the deviation of the osteotomy direction in degrees for either experienced or inexperienced surgeons.

### Facet Joint Violation

**Figure 7.**
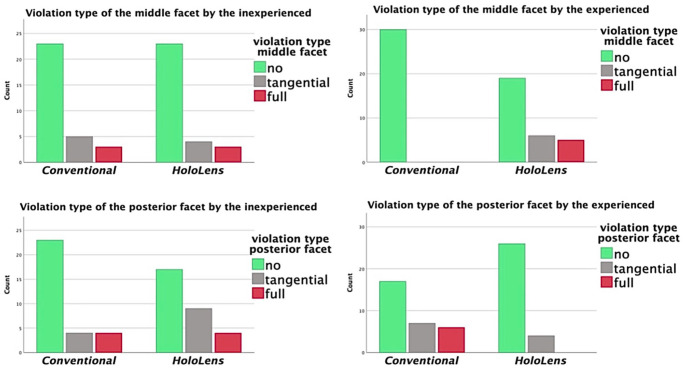
No statistically significant difference in facet violation for inexperienced surgeons (left side). Using HoloLens, experienced surgeons avoided violation of the posterior facet, while violating the middle facet more often instead (right side).

No statistically significant difference was found in the violation of the middle or posterior facets between AR-guided and conventional osteotomies for either the entire study population or the inexperienced surgeons (Figure 7).

However, the experienced surgeons were found to tangentially violate the middle facet more often using AR guidance compared with the conventional method, and this difference was statistically significant (*P* = .0093). More crucially, AR guidance helped the experienced surgeons avoid full violation of the posterior facet, with 6 full violations in the conventional group and none in the AR-guided group (*P* = .011) (Figure 7).

**Figure 8.**
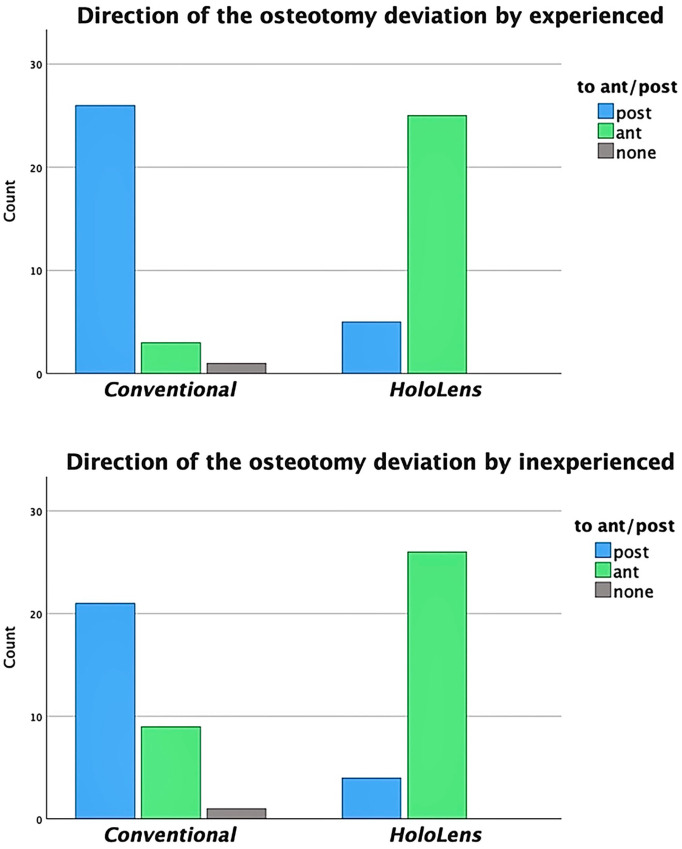
Statistically significant tendencies of the osteotomy deviation from the ideal osteotomy plane in experienced and inexperienced group: direction posterior in conventional and direction anterior in AR-guided osteotomies.

A statistically significant difference was found in the deviation of the osteotomy direction between the 2 methods performed by the experienced and inexperienced group (*P* < .05). Both experienced and inexperienced surgeons showed a clear tendency of posterior deviation from the ideal osteotomy plane in conventional osteotomies. Most of the AR-guided osteotomies by experienced and inexperienced surgeons, on the other hand, demonstrated anterior deviation from the ideal osteotomy plane (Figure 8).

In comparison to the experienced surgeons, the inexperienced surgeons had a higher rate of middle facet injury with the conventional method (3 fully and 5 tangentially violated out of 30) and a higher rate of posterior facet injury with AR-guided osteotomy (4 fully and 9 tangentially violated out of 30). Both of these differences were statistically significant (*P* = .005 and .021, respectively).

## Discussion

The results of the present study suggest that the use of AR guidance during osteotomies leads to a statistically significant improvement in the accuracy of the osteotomy entry point. The mean deviation from the ideal entry point was found to be 1.8 ± 1.3 mm (95% CI 1.48, 2.12) with AR guidance compared to 2.5 ± 1.5 mm (95% CI 2.17, 2.91) without, and experienced surgeons showed a statistically significant improvement in accuracy with AR guidance compared to conventional methods (*P* = .035). However, the results did not show a statistically significant difference in the deviation of the exit point or the osteotomy direction.

The comparison between conventional and AR-guided osteotomies did not yield a statistically significant reduction in the facet violation rate. However, a closer examination of the outcomes in the experienced group highlights the potential benefits of AR guidance in preventing posterior facet violations. In the conventional osteotomy group, 6 of 30 posterior facets were fully violated, whereas no full violations were observed in the AR-guided group. This is believed to be due to the tendency of both experienced and inexperienced surgeons to direct the osteotomy from posterior to anterior using AR guidance, which prevented the saw blade from accidentally violating the posterior facets. The visual guidance provided by the holographic osteotomy plane served as a barrier that alerted the surgeon to readjust the direction of the osteotomy if the saw blade strayed too far.

However, this same tendency toward anterior-to-posterior osteotomy direction also resulted in a higher rate of tangential violations to the biomechanically less vital middle facet, as compared to the conventional group. This issue of variation in osteotomy direction could be addressed through a revision of the holographic osteotomy plane design. The introduction of two separate holographic planes to guide the surgeon in placing the blade exactly between them would mitigate this issue and also provide additional visual barriers to protect both the middle and posterior facets. Further research is needed to evaluate these modifications and their potential impact on AR-guided osteotomies.

The influence of surgical experience on the rate of facet violations was analyzed and found to be a significant factor. In the conventional osteotomy group, experienced surgeons never fully violated the middle facet, whereas inexperienced surgeons incurred 3 full and 5 tangential violations. In the AR-guided group, experienced surgeons never fully violated the posterior facet, while inexperienced surgeons had 4 full violations. The key finding here is that AR guidance helped experienced surgeons to prevent full violations of the posterior facet, which is a biomechanically vital articular surface owing to its role as a major loadbearing area.^10^ This highlights the potential of AR to not only provide guidance but also protect critical structures during surgical procedures. In case of the inexperienced, we assume that they were rather distracted by additional visual information from AR in the setting of absent osteotomy skills. In other similar studies, such as for example by Viehöfer et al,^24^ the inexperienced rather benefited from AR guidance. The busy visual environment might have also led to deviation of the saw blade, leading to no improvement of the exit point with AR in the experienced group.

The limitations of our study should be noted. First, the anatomy of the facets varies among patients. Our AR guidance was based on a single model and was used for 120 foot bone models. In actual clinical practice, AR guidance with an ideal osteotomy plane should be planned meticulously based on the specific anatomy of the facets. Second, we intentionally did not have a stop planned before penetrating the medial calcaneal cortex, and that is likely different from how some surgeons prefer to perform this opening-wedge osteotomy. Third, the alignment of the hologram with the foot should be improved further. Despite our diligent manual marking of the predetermined landmarks, misalignment may still occur. In such cases, error feedback from the AR system would be highly beneficial.

The results of this study highlight the potential benefits of combining AR guidance and surgical experience in achieving optimal accuracy during H-O procedures. Further research, such as a study conducted on cadaver feet with varying facet anatomy, is needed to definitively confirm the accuracy and superiority of AR-guided H-O compared with conventional techniques. Despite its potential to enhance intraoperative navigation and minimize the need for 2-dimensional intraoperative fluoroscopy, it is important to acknowledge the limitations and challenges associated with AR technology. Improved automatic alignment of the hologram and individualized preoperative AR planning for each patient’s unique anatomy are crucial for maximizing the efficacy of AR in hindfoot surgery and other procedures with complex anatomy and vulnerable structures at risk. The cost and time required for preoperative AR planning must also be considered. Although the starting price of HoloLens is currently $3500,^19^ there is an additional cost of a surgical planning software. Ultimately, the use of AR in surgery has the potential to contribute to patient, surgeon, and operating room staff safety by providing real-time 3D visualization and minimizing radiation exposure. In the setting of a well-tested and approved AR guidance for a specific procedure, fluoroscopy could be completely eliminated or used only for a postoperative control. In the future, this could be the case for many foot and ankle procedures including placement of internal fixation.

In conclusion, application of AR guidance during H-O was associated with improved accuracy for experienced surgeons, demonstrated by a better accuracy of the osteotomy entry point. More crucially, AR guidance prevented full violation of the posterior facet in the experienced group. In contrast, AR did not provide any benefit to the inexperienced. Further research is needed to address limitations and test this technology on cadaver feet. Ultimately, the use of AR in surgery has the potential to improve patient and surgeon safety while minimizing radiation exposure.

## Supplemental Material

sj-pdf-1-fai-10.1177_10711007241237532 – Supplemental material for Conventional vs Augmented Reality–Guided Lateral Calcaneal Lengthening Simulated in a Foot Bone Model Supplemental material, sj-pdf-1-fai-10.1177_10711007241237532 for Conventional vs Augmented Reality–Guided Lateral Calcaneal Lengthening Simulated in a Foot Bone Model by Maksym Polt, Arnd F. Viehöfer, Fabio A. Casari, Florian B. Imhoff, Stephan H. Wirth and Stefan M. Zimmermann in Foot & Ankle International
